# Prokaryotic Collagen-Like Proteins as Novel Biomaterials

**DOI:** 10.3389/fbioe.2022.840939

**Published:** 2022-03-17

**Authors:** Jonathan Picker, Ziyang Lan, Srishtee Arora, Mykel Green, Mariah Hahn, Elizabeth Cosgriff-Hernandez, Magnus Hook

**Affiliations:** ^1^ Center for Infectious and Inflammatory Diseases, Institute of Biosciences and Technology, Texas A&M, Houston, TX, United States; ^2^ Department of Biomedical Engineering, The University of Texas at Austin, Austin, TX, United States; ^3^ Department of Biomedical Engineering, Rensselaer Polytechnic Institute, Troy, NY, United States

**Keywords:** collagen, prokaryotic collagen-like protein, streptococcal collagen-like protein, integrin, integrin-targeting biomaterials, biomaterials, hydrogel

## Abstract

Collagens are the major structural component in animal extracellular matrices and are critical signaling molecules in various cell-matrix interactions. Its unique triple helical structure is enabled by tripeptide Gly-X-Y repeats. Understanding of sequence requirements for animal-derived collagen led to the discovery of prokaryotic collagen-like protein in the early 2000s. These prokaryotic collagen-like proteins are structurally similar to mammalian collagens in many ways. However, unlike the challenges associated with recombinant expression of mammalian collagens, these prokaryotic collagen-like proteins can be readily expressed in *E. coli* and are amenable to genetic modification. In this review article, we will first discuss the properties of mammalian collagen and provide a comparative analysis of mammalian collagen and prokaryotic collagen-like proteins. We will then review the use of prokaryotic collagen-like proteins to both study the biology of conventional collagen and develop a new biomaterial platform. Finally, we will describe the application of Scl2 protein, a streptococcal collagen-like protein, in thromboresistant coating for cardiovascular devices, scaffolds for bone regeneration, chronic wound dressing and matrices for cartilage regeneration.

## Introduction

Collagen is the most abundant protein found in humans and other animals (Shoulders and Raines 2009; Pallela, Ehrlich, and Bhatnagar 2016). It is a major component of the extracellular matrix (ECM) that provides structural stability (Chang and Buehler 2014). Collagen also regulates cell and tissue biology by interacting with various cellular receptors and other extracellular components (Frantz, Stewart, and Weaver 2010). Its ubiquity within every animal signifies its importance in the formation and maturation of an organism; thus, it has been the target of many studies from the earlier 1900s through today (Siegfried 1902). These studies have improved the understanding of the structural features of collagens including the Gly-X-Y repeat necessary for its characteristic triple helical structure (Qiu et al., 2021; Chen, Chen, and Horng 2011). Understanding the sequence requirement for the triple helix formation led to the discovery that organisms other than animals such as prokaryotes can produce collagen-like proteins as well (Xu et al., 2002; Lukomski et al., 2000). Many different species of bacteria have been shown to produce collagen-like proteins, referred in this review article as prokaryotic collagen-like proteins, though the predominantly studied proteins come from the *Streptococcus* and the *Bacillus* families of bacteria (Lukomski et al., 2017; Qiu et al., 2021). The discovery of these prokaryotic collagen-like proteins has provided a new experimental platform. The streptococcal collagen-like protein, Scl2.28, provides a “blank slate” for protein engineering studies due to its inability to bind to any known ligand (Xu et al., 2002). The ability to recombinantly produce collagen-like molecule, based on Scl2.28 in *E. coli* has enabled the usage of collagen-like protein for multiple engineering applications from endothelialization of vascular grafts to bone regeneration (Cosgriff-Hernandez et al., 2010; Parmar et al., 2016a; Parmar et al., 2016b; Becerra-Bayona et al., 2018; Post et al., 2019).

In this review, we will first discuss the properties of mammalian collagen and provide a comparative analysis of mammalian collagen and prokaryotic collagen-like proteins. We will then review the use of prokaryotic collagen-like proteins to both study the biology of conventional collagen and develop a new biomaterial platform. The application of prokaryotic collagen-like proteins in integrin-targeting hydrogels in a range of biomedical applications will be highlighted. Overall, this review lays a foundation for utilizing recombinant bacterial collagen to perform fundamental studies on the structure and function of mammalian collagen and pursue advanced biomaterials that orchestrate specific cellular behaviors.

## Human and Mammalian Collagen

Based on genetic sequence, 28 different types of collagens have been documented in humans (Söderhäll et al., 2007). In addition, several proteins, such as C1q, ficolins, and adiponectin contain minor collagenous domains but are not usually regarded as collagens since their functions and overall structures are not related to conventional collagens (Ricard-Blum, 2011).

### Structure of Collagen

**FIGURE 1:**
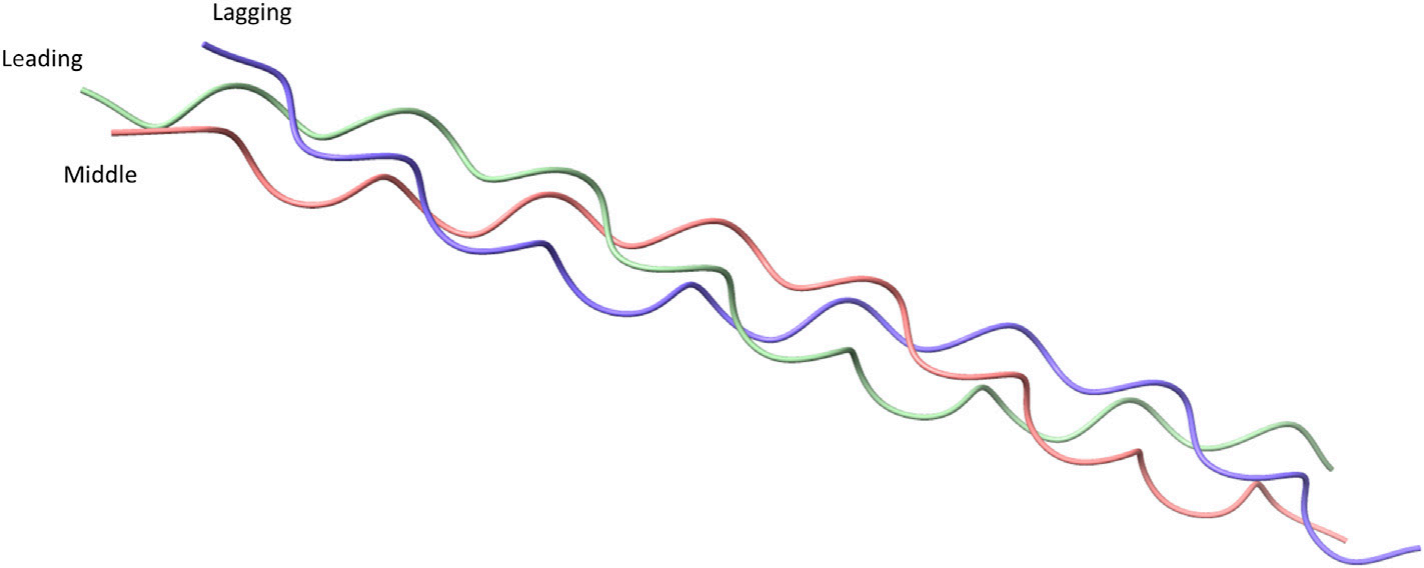
Crystal structure of a collagen peptide with the sequence (PPG)_10_, accessed from **PDB ID: 1K6F**. The crystal structure shows the structure of the triple helical collagen. Three individual peptides are shown with green representing the leading strand of collagen, pink representing the middle strand, and blue representing the lagging strand.

Collagens are defined by the presence of a unique Gly-X -Y tripeptide repeat where X is usually a proline and Y is usually hydroxyproline, which is required by eukaryotic collagen for stable triple-helix formation. Each collagen molecule is made up of three individual α-chains where each α-chain has the structure of a left-handed polyproline Type II-like helix. The three α-chains together form a tightly packed right-handed helix with a 1.5 nm diameter (Figure 1) (Lodish et al., 2000; Berisio et al., 2002; Adzhubei, Sternberg, and Makarov 2013; Qiu et al., 2021). Supercoiling of the individual α-chains in the trimeric structure leads to a rise of 2.9Å per residue and 3.33 residues per turn of the individual α-chains (Persikov et al., 2000). This packing requires each strand to rotate such that the glycine residues lie within the center (Lodish et al., 2000; Berisio et al., 2002). As anything larger than a glycine residue in the center would not fit in the trimeric helix due to steric hindrance (Chen, Chen, and Horng 2011).

Collagen molecules can be formed by three identical α-chains or by a mixture of two to three different α-chains. The α-chain composition of different collagens is reviewed elsewhere (Birk and Peter, 2011; Ricard-Blum, 2011). Formation of homotrimer or heterotrimer is directed by a 20 to 250 amino acids long non-collagenous domain (Snellman et al., 2000; Boudko, Engel, and Bächinger 2012; Boudko and Bächinger 2016; Sharma et al., 2017). When collagen α-chains align to form a trimer, a staggered formation is produced where each α-chain has glycine residues in the center of the structure, with the leading strand being the first α-chain to have glycine on the inside of the helix. The middle and lagging strands are the second and third α-chain with the glycine on the inside of the helix, respectively (Figure 1). The stagger of α-chains must be specifically controlled because an incorrect stagger prevents appropriate interactions from occurring (Boudko and Bächinger 2016; 2012).

### Collagen Superstructures

These triple helical collagen molecules then assemble into different supermolecular structures leading to the fibers and networks found in mammalian tissues. Based on the supermolecular structure and assemblies, collagen can be divided into different classes: fibrillar collagens, anchoring fibrils, beaded-filament forming collagens, network-forming collagen, fibrillar associated collagens with interrupted triple helices (FACIT), membrane associated collagen with interrupted triple helices (MACIT), multiplexins, and uncategorized collagens (Kucharz 1992; Ottani et al., 2002; Kielty and Grant 2003; Birk and Peter, 2011; Ricard-Blum, 2011). These different classes are reviewed below briefly.

Fibrillar collagens, which includes Type I, II, III, V, XI, XXIV, XXVII Collagens, is the most prominent class of collagen (Birk and Peter, 2011). Fibrillar collagens represent the largest percentage of collagen present in humans and are dominant structural components in the extracellular matrix in mammals (Gelse, Pöschl, and Aigner 2003). As the name suggests, fibrillar collagens form fibrils, which in turn form fascicles, and fascicles then assemble to form fibers (Fratzl 2003).

The non-fibrillar collagens form a variety of superstructures, encompassing all of the classes of collagen except the fibrillar collagen class. The beaded filament-forming collagen include Type VI, Type XXVI and Type XXVIII collagens, non-covalently assembling into thin and beaded filaments where globular domains appear as “beads” (Knupp and Squire 2003; Birk and Peter, 2011). The network-forming collagens, which includes Types IV, VIII, and X collagens, form non-covalent networks similar to the beaded filament-forming collagens but in a diamond or a hexagonal shape (Knupp and Squire 2003; Birk and Peter, 2011). Type IV Collagen; the archetype of network-forming collagens, is found in basement membrane, and is essential for basement membrane function (Khoshnoodi, Pedchenko, and Hudson 2008; Sand, Genovese, and Karsdal 2016). Anchoring fibril collagen i.e. Type VII Collagen, associates laterally via C-terminal of collagen monomers while the N-terminal attaches to the basement membrane (Chung and Uitto 2010; Birk and Peter, 2011). FACITs include Type IX, XII, XIV, XVI, XIX, XXI, and XXII Collagens, and interact with larger fibrillar collagens, such as Type I Collagen or Type II Collagen (Birk and Peter, 2011). In addition, Type IX Collagen can also incorporate into the fibrillar collagens of cartilage (Birk and Peter, 2011). MACITs, also known as membrane collagens, includes Type XIII, XVII, XXIII, and XXV collagens, and integrate into the cell membrane and anchor the cell to the respective basement membrane (Birk and Peter, 2011; Ricard-Blum, 2011). Multiplexin collagens, Type XV and XVIII, are integrated into the basement membrane (Birk and Peter, 2011).

### Post-Translational Modifications of Collagen

All mammalian collagens undergo post-translational modifications that happen both before and after collagen is secreted from the cell. Individual α-chains are post-translationally modified before trimerization and secretion from the cell. These post-translation modifications include: 1) hydroxylation of some proline residues into 3-hydroxyproline or 4-hydroxyproline by prolyl-3-hydroxylase or prolyl-4-hydroxylase respectively, 2) hydroxylation of some lysine residues into hydroxylysine by lysyl hydroxylase, and 3) glycosylation of some of the hydroxylysine residues by hydroxylysyl galactosyltransferase and galactosylhydroxylysyl-glucosyltransferase (Gelse, Pöschl, and Aigner 2003). These post-translational modifications of individual α-chains cannot occur once the trimeric structure has been formed (Patino et al., 2002). Next, C-terminal non-collagenous regions are linked together by disulfide bonds catalyzed by protein disulfide isomerase to ensure correct strand alignment and trimer formation (Gelse, Pöschl, and Aigner 2003; Seibel, Robins, and Bilezikian 2006). Once the trimeric collagen molecule is assembled, it can be secreted out of the cell. Once outside the cell, the N-terminal and C-terminal pro-peptides of collagen molecule are cleaved by procollagen N-proteinase and procollagen C-proteinase, respectively (Patino et al., 2002). Finally, after the cleavage of N- and C-terminal propeptides, the collagen molecule is cross-linked to other collagen molecules through hydroxylysines, forming the fibril superstructure (Gelse, Pöschl, and Aigner 2003).

## Prokaryotic Collagen-Like Proteins

For a long time, it was believed that non-eukaryotes cannot produce collagen. However, in the year 2000 *Streptococcus pyogenes*, a Gram-positive bacteria, was discovered to produce collagen-like protein named Streptococcal collagen-like protein A (SclA) (Lukomski et al., 2000). Later discoveries revealed that *S. pyogenes* encodes two genes, which produce two different proteins containing a collagen-like region: SclA and SclB (Whatmore 2001). These proteins have since been renamed, and are known today as Scl1 and Scl2 (Humtsoe et al., 2005). At the time, this discovery was surprising because it was believed that the characteristic triple helix cannot be formed without post-translational modification of proline into hydroxyproline, a post-translational mechanism specific to eukaryotes. Yet, Scl1 and Scl2 proteins were confirmed to have triple helical structure using circular dichroism (CD) where peak around 220 nm was observed (Xu et al., 2002). The peak at 220 nm is characteristic for the collagen triple-helical structure. In addition, rotary shadowing microscopy of Scl1 and Scl2 proteins showed a lollipop-like structure similar to human collagens (Xu et al., 2002; Adzhubei, Sternberg, and Makarov 2013). Since then, other bacterial species such as, *Bacillus anthracis, Clostridium difficile, Clostridium perfringens*, *Legionella pneumophila,* and *Burkholderia* have been shown to produce collagen-like proteins (Rasmussen, Jacobsson, and Björck 2003; Yu et al., 2014; Bachert et al., 2015; Schnicker and Dey 2016; Ellison et al., 2020). Both pathogenic and non-pathogenic soil bacteria, like *Bacillus amyloliquefaciens*, produces collagen-like proteins (Yu et al., 2014).

Admittedly in 1990, i.e., prior to the discovery of prokaryotic collagen-like proteins, viruses were discovered to produce collagen-like proteins, though these have been predominantly recognized in bacteriophages (Bamford and Bamford 1990; Rasmussen, Jacobsson, and Björck 2003). Some giant viruses even produce collagen and have the ability to hydroxylate and glycosylate their collagen, namely the mimivirus which infects amoebas (Luther et al., 2011; Yamada 2011). However, the bacteriophage collagens are only about 18 residues found within capsid proteins, and the collagens found within giant viruses rely on eukaryotic systems to be produced (Bamford and Bamford 1990). Consequently, the corresponding viral genes may not lend themselves to direct manipulation and expression in prokaryotic expression systems. These viral genes will not be further discussed in this review.

### Domain Organization of Prokaryotic Collagen-Like Proteins

**FIGURE 2:**
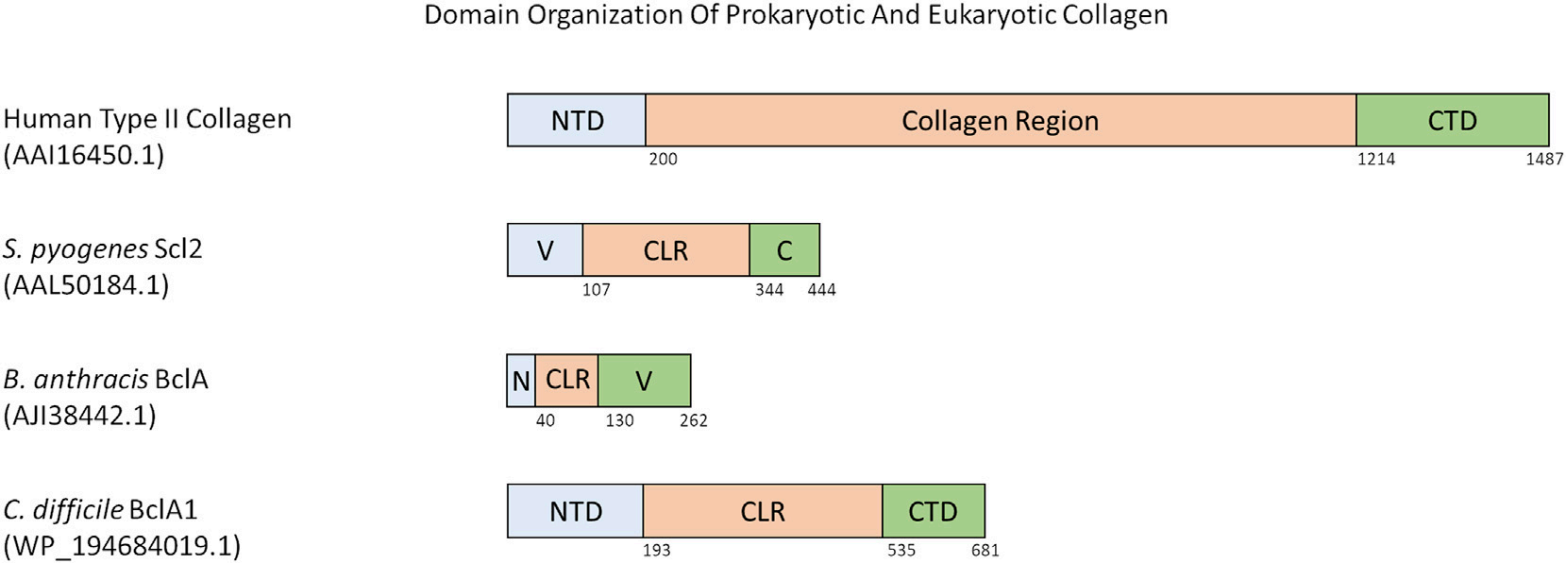
Domain organization of various eukaryotic and prokaryotic collagens. All numbers in parenthesis below species name and protein name is the accession number for the protein as found in the NCBI Protein database. Numbers below the domains indicate the last residue of that domain. Abbreviations are as follows: NTD = N-terminal Domain, CTD = C-terminal Domain, V = Trimerization Domain, C = C-terminal domain and anchor to cell membrane, N = N-terminal Domain and anchor to cell membrane, CLR = Collagen-like Region.

There are a few different domain organization formats of prokaryotic collagen-like proteins. Such as, the prototype Scl proteins from *S. pyogenes*, have an N-terminal variable domain that is able to nucleate trimerization of the collagen-like region with the characteristic Gly-X-Y repeats followed by a C-terminal non-collagenous repeat with motifs required for cell wall anchoring (Lukomski et al., 2017). The variable domain is significantly different between Scl1 and Scl2, and differs between M-types of *S. pyogenes* (Caswell et al., 2010). Both Scl1 and Scl2 proteins contain a variable number of Gly-X-Y repeats and sequence in their collagen-like region. For example, Scl1 protein from *S. pyogenes* M1 serotype contains 50 Gly-X-Y repeats whereas Scl2 from serotype M28 contains 79 Gly-X-Y repeats (Xu et al., 2002; Lukomski et al., 2017; Qiu et al., 2021) (Figure 2).

Some prokaryotic collagen-like proteins have different domains flanking the collagen-like region. For example, the collagen-like proteins Bucl3 and Bucl8 from *Burkholderia* bacteria also contain the Talin-1 domain for binding to integrins, and outer membrane efflux protein domains to form an efflux channel in the outer membrane of the bacteria (Bachert et al., 2015; Grund et al., 2020). *S. pneumoniae* protein PclA has a collagen-like region interspersed with a non-collagen domain and other small interruptions, and a large C-terminal domain containing an LPXTG motif (Paterson et al., 2008). Within the *Bacillus* genus, a variety of collagen-like proteins have been reported (Qiu et al., 2021; Leski et al., 2009). These are named as Bcl proteins with a corresponding letter at the end e.g. BclA, BclB (Parenteau-Bareil, Gauvin, and Berthod 2010). Bcl proteins are composed of a N-terminal domain, a linker region present in some Bcl proteins, a collagen-like region of 27 to 1,158 residues, and finally a C-terminal domain that can have different predicted folds (Figure 2) (Leski et al., 2009). The similarly named BclA1, BclA2, and BclA3 found in *C. difficile* has a basic domain structure of a N-terminal domain between 5 and 193 residues, a collagen-like region with 354–422 residues, and a C-terminal domain with 131–148 residues (Figure 2) (Pizarro-Guajardo et al., 2014). Each N- and C-terminal domain of the BclA1, BclA2, and BclA3 proteins are currently not well studied and thus have unknown structures within *C. difficile*.

In summary, prokaryotic collagen-like proteins have diverse domain organization but generally contain a collagen-like region flanked by non-collagenous N-terminal and C-terminal domains. It is noticeable that a similar domain organization is also seen for fibrillar eukaryotic collagens (Figure 2).

### Structure of Collagen-Like Regions

Similar to vertebrate collagen, prokaryotic collagen-like proteins contain Gly-X-Y tripeptide repeats, where glycine is required every third residue. The X and Y positions of prokaryotic collagen-like proteins can be occupied by any amino acids, but some residues are preferred (see below). Despite the lack of hydroxyproline, prokaryotic collagen-like proteins form triple helical structures that show the characteristic 220 nm peak in CD (Xu et al., 2002). In addition, the ratio of intensity of the peak at 220 nm over intensity of the peak at 198 nm for the collagen-like region of Scl 2.28 from *S. pyogenes* is similar to that of mammalian collagen, indicating that the triple helix is fully formed. The collagen-like region of Scl 2.28 from *S. pyogenes* forms a triple-helix with a diameter of 4–5 nm (Yoshizumi et al., 2009). Similar to mammalian collagen, the collagen-like region of prokaryotic collagen-like proteins is resistant to trypsin digestion (Xu et al., 2010). Prokaryotic collagen-like proteins also appear as lollipop-like structures in rotary shadowing microscopy similar to human collagens (Xu et al., 2002; Adzhubei, Sternberg, and Makarov 2013).

Most bacteria cannot produce hydroxyproline. So how do bacteria produce stable collagen-like structures? Collagen-like region of *S. pyogenes* Scl proteins contain a large number of polar or charged residues in X and Y positions (Mohs et al., 2007). The most common residue in bacterial collagens present in the X position is proline, similar to vertebrate collagen, whereas threonine is the predominant residue in the Y (Rasmussen, Jacobsson, and Björck 2003; Ghosh et al., 2012). Polar residues in the Scl protein interact with the water molecules to create an extensive network of water-mediated hydrogen bonding, similar to the role that hydroxyproline has in eukaryotic collagen. Such networks are called hydration networks. It stabilizes the triple helix and provides Scl2 with a higher thermal melting temperature of 36°C (Mohs et al., 2007). NMR studies of rat-tail tendon and crystal structures of synthetic collagen peptides have shown that a hydration network occurs in human collagen as well (Migchelsen and Berendsen 1973; Mohs et al., 2007). Mohs et al. demonstrated the effect of hydration network on bacterial collagens by measuring the thermal melting temperature shift that occurs when bacterial collagen is placed in a neutral and an acidic buffers, respectively (Mohs et al., 2007). Scl2.28 protein, which has a low proline percentage but higher percentage of lysine and aspartic acid residues, has a melting temperature of 35.6°C at neutral pH and shifts to 24.2°C at 2.2 pH (Mohs et al., 2007). This large shift is believed to be caused by the loss of charge on aspartic acid and glutamic acid residues in Scl2.28, thus preventing the formation of an extensive hydration network (Mohs et al., 2007).

For those in the phylum Firmicutes, which includes *Bacillus* and *Streptococcus*, the collagen-like regions of the proteins are composed of approximately 16.42% threonine, 10.09% proline, 9.17% alanine, and 7.89% glutamine residues (Qiu et al., 2021). Firmicutes have the highest total ratio of threonine and glutamine residues, revealing the importance of hydration network formation for these collagen-like proteins to form stable triple helices (Qiu et al., 2021). Other phyla of bacteria have higher percentages of alanine or proline residues within the collagen-like regions (Qiu et al., 2021).

### Post-Translation Modifications of Prokaryotic Collagen-Like Proteins

Prokaryotes are unable to generate all the post-translation modifications observed in vertebrates. While bacteria may not be able to employ all post-translational modifications required for collagen formation in mammals, they do employ some post-translational modifications that are thought to lead to stable triple-helical collagens (Sylvestre, Couture-Tosi, and Mock 2002; Dell et al., 2011). *B. anthracis* produces a prolyl-4-hydroxylase enzyme that converts proline to hydroxyproline in synthetic peptides, implying the ability for *B. anthracis* to improve the stability of their collagen proteins by introducing hydroxyproline. Although the *bacillus* enzyme does not work as consistently as eukaryotic prolyl-4-hydroxylase (Schnicker and Dey 2016). BclA is also known to be glycosylated in *B. anthracis* and *B. cereus* spores (Tan and Turnbough 2010; Thompson et al., 2012). It is believed this glycosylation of BclA protein improves hydration network formation and interaction because the sugar moiety is able to interact with multiple water molecules in solution (Sylvestre, Couture-Tosi, and Mock 2002; Boydston et al., 2005; Yu et al., 2014). Similarly, BclA1 protein from *C. difficile* has been shown to be glycosylated (Pizarro-Guajardo et al., 2014). Additionally, the *C.* difficile BclA1 protein is cleaved from a ∼72 kDa protein into a 33 kDa protein, though not much more is known about this process (Díaz-González et al., 2015).

### Function of Prokaryotic Collagen-Like Proteins

Bacterial collagen-like proteins can have very different functions. Some of the functions are in the non-collagenous domains while others depend on the motifs in the collagen-like region. For example, Scl1 and Scl2 proteins from the M1 serotype of *S. pyogenes* can intertwine with wounded (denatured) human collagen found within wounded tissue, aiding in invasion and adherence to the wounded site during the infection process (Ellison et al., 2020). In addition, Scl1 protein binds to fibronectin and integrins (α2β1 and α11β1) and mediates bacterial adherence to the wound microenvironment, intracellular invasion, and evasion of the immune system (Caswell et al., 2008, 2010; Oliver-Kozup et al., 2011; Oliver-Kozup et al., 2013; Lukomski et al., 2017). The *S. pneumoniae* PclA protein has been shown to play a role in adherence and invasion of host cells (Paterson et al., 2008). BclA1 from *C. difficile* is required for colonization and infection in animals (Pizarro-Guajardo et al., 2016). *B. anthracis* BclA protein is known to play a role in stabilizing the exosporium of the spore and has reported melting temperatures of up to 95°C due to the very stable C-terminal domain (Boydston et al., 2005). Collagen-like protein expressed by *B. amyloliquefaciens*, a bacteria found in soil, enables it to adhere to plant roots where they promote plant growth (Zhao et al., 2015). Two of the four collagen-like proteins in *B. amyloliquefaciens* have also been reported to be present within the flagella of the cell and play a role in motility (Zhao et al., 2016).

## Structural Similarities and Differences Between Eukaryotic and Prokaryotic Collagens

Both vertebrate and prokaryotic collagen-like proteins have Gly-X-Y repeats in their primary sequence. Despite the absence of hydroxyproline in prokaryotic collagen-like proteins, its’ individual chains form Type II helices as observed in mammalian collagen and form a triple-helical structure characteristic of mammalian collagen (Xu et al., 2002). Although mammalian collagen can form superstructures, such self-assemblies have not been observed for collagen-like proteins found in prokaryotes (Table 1). Proline occupies approximately one-third of the X position within the Gly-X-Y repeat of both human collagen and collagen-like proteins in Gram-positive bacteria (Ghosh et al., 2012). Human collagen often contains hydroxyproline in the Y position whereas Gram-positive bacteria often use threonine at the Y position (Ghosh et al., 2012) (Table 1). The differences between mammalian and bacterial collagens are summarized in Table 1.

**TABLE 1:** Characteristics of eukaryotic collagens and prokaryotic collagen-like proteins.

	Eukaryotic collagen	Prokaryotic collagen-like protein	References
Domain Organization	Has a N-terminal domain, a collagen region, and a C-terminal domain	Has a N-terminal domain, a collagen region, and a C-terminal domain	Qiu et al., 2021
	Might have some interruptions within the collagen region	Might have some interruptions within the collagen region	Bachert et al., 2015
			Paterson et al., 2008
			Lukomski et al., 2017
			Leski et al., 2009
			Tan and Turnbough 2010
			Pizarro-Guajardo et al. (2014)
Residue Composition of collagen region (X/Y)	Gly-X-Y (Percentages based on human collagen)	Gly-X-Y	Rasmussen et al. (2003)
	X: 31.1% Proline (fibrillar), 24.6% Proline (non-fibrillar)	X: 31.0% Proline (Gram-positive), 19% Proline (all bacteria)	Ghosh et al., 2012; Hynes 2012
	Y: 33.5% Proline/hydroxyproline (fibrillar), 42.2% Proline/hydroxyproline (non-fibrillar)	Y: 48.3% Threonine (Gram-positive), 31.6% Threonine (all bacteria)	
Post-translational modification	Hydroxylation of proline to hydroxyproline and lysine to hydroxylysine, glycosylation of hydroxylysine residues, removal of N-terminal and C-terminal pro-peptides, and cross-linking of lysine and hydroxylysine residues	In most cases, there is no hydroxylation of proline to hydroxyproline, no glycosylation of the collagen structure, and no cleavage of the protein	Schnicker and Dey 2016
		A limited number of bacteria have their own post-translational modifications, allowing for hydroxylation, glycosylation, or cleavage of proteins	Tan and Turnbough 2010
			Thompson et al., 2012
			Gelse et al. (2003)
Function	Provides mechanical properties (load bearing, tensile strength, and torsional stiffness), cell signaling either directly or through other ECM components, filtration, adhering materials together	Adhesion to specific environments, formation of biofilm, internalization into host, evasion of immune system, use for motility, and spore formation	Qiu et al., 2021
			Gelse et al. (2003)
			Bachert et al., 2015
			Paterson et al., 2008
			Lukomski et al., 2017
			Leski et al., 2009
			Tan and Turnbough 2010
			Pizarro-Guajardo et al., 2014
			Zhao et al., 2015
			Zhao et al., 2016
			Hynes 2002
			Paten et al., 2019
			Shaw and Olsen 1991
			Izzi et al., 2020
			Brazel et al. (1988)
Location	Present within the matrix, with some cellular receptor binding (such as integrin)	N-terminal or C-terminally bound to bacteria cell wall, Bucl8 has two outer transmembrane domains	Qiu et al., 2021
	A few collagens can be found integrated within cell membranes		Bachert et al., 2015
			Lukomski et al., 2017
			Izzi et al. (2020)
Ability to form superstructures	Fibrillar collagen can come together to form larger fibrils, fascicles, and tendons	Unable to form larger superstructures, none are currently known	Qiu et al., 2021
			Gelse et al. (2003)
			Boydston et al., 2005
			Canty and Kadler (2002)

## Using Prokaryotic Collagen-Like Protein to Investigate Eukaryotic Collagen Biology

Various tools have been used to investigate the specific interaction sites on eukaryotic collagen, e.g. imaging of complexes for locating binding sites, cyanogen bromide to cleave the collagen into smaller peptides, synthetic collagen peptides to determine residues involved in binding to a target, and there is production of recombinant and mammalian collagen using yeast and transgenic plants (Farndale et al., 2008; Brodsky and Kaplan 2013; Farndale 2019; Fertala 2020). These techniques each have their unique strengths and weaknesses. Cyanogen bromide cleavage might lead to cleavage of the binding site or generate too large of a collagen fragment to accurately locate the binding residues. The success of an imaging approach depends on the resolution and availability of reference structures (Perumal, Antipova, and Orgel 2008; Sweeney et al., 2008; Orgel et al., 2009). Synthetic collagen peptide works effectively for shorter sequences but has difficulty if the binding sequence is large. Although synthetic collagen peptides provide the opportunity to insert peptide binding sequences into a triple helical framework, binding regions larger than nine residues cannot be investigated (Farndale 2019). There have been limited advancement in producing recombinant collagen from yeast and transgenic plants; however, both of these routes are significantly more time-intensive and expensive than utilizing *E. coli* for production of bacterial recombinant collagen-like proteins (Brodsky and Kaplan 2013; Fertala 2020).

Prokaryotic collagen-like protein provides a novel strategy for investigating interactions of vertebrate collagen with other proteins. Prokaryotic collagen-like proteins can serve as a “host” to a “guest” eukaryotic collagen sequence. Collagen-like region from *S. pyogenes* protein Scl2.28 comprising 79 Gly-X-Y repeats does not interact with known collagen binding partners in vertebrates and has thus been named a “blank slate” (Qiu et al., 2021; Cereceres et al., 2015; Xu et al., 2002). This “blank slate” collagen can act as a “host” to “guest” sequence from vertebrae collagen sequences. This “host-guest” collagen, referred to as recombinant bacterial collagen in this review, can be used to insert sequences from human collagen allowing for the discovery of longer binding sequences or investigating multiple binding sequences within one protein. An et al. inserted 24 amino acids into blank slate Scl 2.28 protein to identify fibronectin binding site in Type II collagen (An et al., 2014). Previously, fibronectin binding site in Type II collagen was defined to be GLO GQR GER (Erat et al., 2009; 2010). Using recombinant collagen, An et al. determined that the binding site for fibronectin in Type II collagen is at least 18 amino acids long (Table 2). Similarly, recombinant bacterial collagen has been shown to be useful in studying the interactions between collagen and integrin, and describing the stability of a Type I Collagen homotrimer against MMP degradation (Humtsoe et al., 2005; An et al., 2014; Mekkat et al., 2018). As can be seen in Table 2, there are still many interactions within eukaryotic collagen that are not well-defined. Recombinant bacterial collagen could be used to identify the residues in vertebrate collagen that are involved in these various interactions.

**TABLE 2:** Binding sites on human collagen to various ECM proteins and cellular receptors. Symbol Definition: * - computational modeling used to define residues, ∼ denotes protease cut site in the protein sequence, † protease cut site was determined using Mass Spectroscopy, “O/P” means that both hydroxyproline and proline have been used and were shown to interact, “x” represents any amino acid unless otherwise stated.

Target protein	Collagen binding site	Investigated using	References
Decorin	“Gx_1_x_2_GDR GEx_3_GP site, where x_1_ = K or A, x_2_ = N, K, S, or P, x_3_ = P or T” *	Full-length Type I Collagen Fibril	Orgel et al. (2009)
Biglycan	Known to share a binding domain with decorin, though exact residues are unknown	Full-length Type I Collagen	Wiberg et al. (2001)
Discoidin Domain Receptor 1	GVMGFO	Synthetic collagen peptide	Xu et al., 2011
Discoidin Domain Receptor 2	GVMGFO +2 other sites	Synthetic collagen peptide	Konitsiotis et al., 2008
			Orgel and Madhurapantula (2019)
Integrin α1β1	GF″O/P″GEN, GF″O/P″GER, GLOGER, GASGER, GVOGEA, and GLOGEN	Synthetic collagen peptide & Prokaryotic collagen-like protein	Xu et al., 2000
			Hamaia et al., 2012
			Knight et al., 2000
			Cosgriff-Hernandez et al. (2010)
Integrin α2β1	GF″O/P″GER	Synthetic collagen peptide & Prokaryotic collagen-like protein	Humtsoe et al., 2005
			Xu et al., 2000
			Knight et al., 2000
			Cosgriff-Hernandez et al., 2010
			Knight et al. (1998)
Integrin α10β1	GFOGER	Synthetic collagen peptide	Hamaia et al. (2017)
Integrin α11β1	GFOGER	Synthetic collagen peptide	Sweeney et al., 2008
			Zhang et al. (2003)
Fibronectin	GLAGQR GIVGLP GQRGER	Prokaryotic collagen-like protein & Synthetic collagen peptide	An et al., 2014
			Erat et al. (2009)
Secreted protein, acidic and rich in cysteine known as “SPARC”	GPOGPS GPRGQO GVMGFO GPKGND GAO	Cyanogen bromide peptides & Synthetic collagen peptide	Giudici et al. (2008)
Osteopontin	Binds Type I Collagen at an unknown binding site	Full-length Type I Collagen	Liu et al., 2007
GpVI	GAOGLR GGAGPO GPEGGK GAAGPO GPO, (POG)_10_	Synthetic collagen peptide	Jarvis et al., 2008
			Horii et al. (2006)
Collagen V	Cross-linked in LPQPPQ EKAHDG GRY region	Full-Length Type I Collagen	Sweeney et al. (2008)
Platelet derived growth factor-BB	Binds Type 1 Collagen at an unknown binding site	Full-length Type I Collagen	Lin et al., 2006
Endo180/uPARAP	GPPGPP GPPGPP GPPSAG FDFSFL PQPPQE KAHDGG RYYRA	Full-length Collagen	Sweeney et al., 2008
			Thomas et al., 2005
			Jürgensen et al. (2011)
Thrombospondin1	Known to bind to Type I, VI, XI Collagen, and to Endostatin	Full-length Collagen	Faye et al. (2009)
THBS1			
Procollagen C-proteinase enhancer-1 (PCPE-1)	Binds to the C-terminal propeptide region, the lysine residues C-terminal of the BMP-1 cut site on α1 Type III Collagen	Type I Procollagen, & recombinant Type III Collagen C-propeptide	Kessler and Adar 1989
			Bourhis et al. (2013)
Bone morphogenetic protein-1 (BMP-1)	YRA ∼ DDA NVVRDR D α1 (I)	Type I and III Procollagen	Kessler et al., 1996
	YRA ∼ DQP RSAPSL R α2 (I)		Lindahl et al. (2011)
	YYG ∼ DEP MDFKIN T α1 (III)		
SMADs	C-terminal propeptide of Type I, III, and V Collagen	Recombinant Type I, III, and V Collagen C-propeptide	Ellis et al. (2003)
Osteoclast-associated receptor (OSCAR)	GxOGPx GFx, GPOGPA GFO, GAOGAS GDR	Synthetic collagen peptide	Zhou et al., 2016
			Barrow et al., 2011
			An and Brodsky (2016)
Bone SialoProtein-2	In the triple-helical region of Type I Collagen, and a binding site within Type XI Collagen KKKSNY TKKKRT LATNSK KKSKM, KKKSNY TKKKRT LATNSK KK	Type I Collagen (treated with pepsin), & peptides of non-collagen regions of Type XI Collagen	Baht et al. (2008)
			Gorski et al. (2021)
Periostin	Binds Type I and V Collagen with an unknown binding site	Full-length Type I and V Collagen	Takayama et al., 2006
			Norris et al., 2007
			Kii and Ito 2017
			Kudo and Kii (2018)
Pigment epithelium-derived factor (PEDF)	KGHRGF SGL, KGHRGY SGL	Synthetic collagen peptide	Sekiya et al., 2011
			Broadhead et al. (2010)
Heparin	GBBGB, where “B” is a base residue	Type I Collagen, Prokaryotic collagen-like protein, & Synthetic collagen peptide	Capila and Linhardt 2002
	GRPGKR GKQGQK, RGTPGK PGPRGQ RGPTGP RGERGPR, GRKGR, GKRGK		Sweeney et al., 1998
			Peng et al., 2014
			Ricard-Blum et al., 2006
			Parmar et al. (2016a)
Heparan-Sulfate	KGHRGF, RGTPGK PGPRGQ RGPTGP RGERGPR	Type I Collagen & Synthetic collagen peptide	Sweeney et al., 2008
			Capila and Linhardt 2002
			Sweeney et al., 1998
			Ricard-Blum et al. (2006)
Leukocyte associated Ig-like receptor-1 (LAIR-1)	GAOGLR GGAGPO GPEGGK GAAGPO GPO	Synthetic collagen peptide	Lebbink et al., 2009 Brondijk et al. (2010)
Heat Shock Protein-47 (HSP47)	xGxRG	Synthetic collagen peptide	Koide et al. (2006)
Fibromodulin	KGHR	Synthetic collagen peptide	Di Lullo et al., 2002
			Kalamajski et al. (2016)
Trypsin-1	Cleaves after lysine or arginine, except when followed by proline	Human collagen, 90% Type I and 10% Type III	Mirigian et al., 2013
	Only works on non-triple helical collagen		Simpson (2006)
Elastase 2	KLK ∼ AR, FVR ∼ NK, And GDR ∼ GL as found in Type XVII Collagen, GPLGIA GITGAR GLA in Type III Collagen	Full-length Type XVII Collagen† & recombinant Type III Collagen	Lin et al., 2012 Williams and Olsen (2009)
Matrix Metalloproteinase-1 (MMP-1)	GPQG ∼ LA GQRGIV GLP	Prokaryotic collagen-like protein, Full-length Type I Collagen	(Birkedal-Hansen et al., 1993; Yu et al., 2012)
MMP-2	GPQG ∼ LA GQRGVV GLP	Full-length Type I Collagen	Sweeney et al., 2008
			Birkedal-Hansen et al. (1993)
MMP-8	GPQG ∼ LA GQRGVV GLP	Full-length Type I Collagen	Sweeney et al., 2008
			Birkedal-Hansen et al. (1993)
MMP-13	G ∼ LAGQR GIVGLO GQRGER, GLOGER GRTGPA GAAGAR	Synthetic collagen peptide	Howes et al. (2014)
Prolyl-4-hydroxylase (P4H)	“X”PG, with “X” being preferred to be Proline	Synthetic collagen peptide	Rapaka et al., 1978
			Kivirikko et al., 1972
			Gorres and Raines (2010)
von Willebrand Factor (VWF)	RGQOGV MGF	Synthetic collagen peptide	Lisman et al. (2006)
Nidogen	Can bind 80 nm away from the C-terminus of Type IV Collagen, and with the ectodomain of Type XIII Collagen	Full-length Type IV Collagen and recombinant Type XIII Collagen ectodomain	Aumailley et al., 1989
			Tu et al. (2002)

The stability of the triple helix structure in different regions of the recombinant bacterial collagen can be controlled by choosing triplets with different predicted melting temperature (Persikov, Ramshaw, and Brodsky 2005). This change in stability of recombinant bacterial collagen can be used to alter the affinity of various interactions (Persikov, Ramshaw, and Brodsky 2005). The nature, number and spacing of specific binding motifs can also be easily manipulated in the recombinant bacterial collagen sequence (Peng et al., 2014). When heparin and integrin binding sites were inserted into blank slate Scl 2.28 protein, recombinant bacterial collagen binding to both proteins could be observed (Peng et al., 2014). This strategy can be used to replicate a collagen sequence that has multiple binding partners and will allow determination of competition for binding to the collagen sequence between multiple ECM components.

While recombinant bacterial collagens as a tool provide a great opportunity to learn more about collagen interactions with other molecules, it has some drawbacks. Recombinant bacterial collagens cannot perfectly replicate eukaryotic collagen yet. The recombinant bacterial collagens produced in *E. coli* do not have the same post-translational modifications as eukaryotic collagen. The lack of collagen monomer cross-linking prevents the formation of larger superstructures found in eukaryotes (Canty and Kadler 2002; Gelse, Pöschl, and Aigner 2003). Additionally, prokaryotic collagen-like proteins are homotrimers, therefore, studying interactions of heterotrimeric eukaryotic collagens remains a challenge.

Some of these issues are currently being addressed, such as the lack of hydroxyproline within prokaryotic collagen-like proteins (Buechter et al., 2003; Pinkas et al., 2011; Schnicker and Dey 2016). Schnicker and Dey expressed *B. anthracis* prolyl-4-hydroxylase enzyme in *E. coli* (and purified) to hydroxylate (P-P-G)_5_ and (P-P-G)_10_ peptides (Schnicker and Dey 2016). While hydroxylation of the peptides using *B. anthracis* prolyl-4-hydroxylase was observed, both the X-position and Y-position prolines were hydroxylated with the Y-position prolines being favored, which might not replicate the exact eukaryote proline hydroxylation (Schnicker and Dey 2016). Using a different approach, Buechter et al. produced a fragment of Type III collagen with hydroxyproline in *E. coli* by supplementing growth media with hydroxyproline and sodium chloride. However, this method does not discriminate in X and Y positions in Gly-X-Y tripeptide (Buechter et al., 2003). Pinkas et al. created a strain of *E. coli* that expresses human prolyl-4-hydroxylase enzyme and allows for the conversion of α-ketoglutarate into l-ascorbate (Pinkas et al., 2011). Using this strain, the authors demonstrated that the proline residues within the P-P-G peptide could be converted into hydroxyproline but less hydroxylation was observed with longer (P-P-G) repeats (Pinkas et al., 2011). While great strides have been made to produce recombinant bacterial collagen with hydroxyproline in *E. coli*, future work is needed to produce longer recombinant bacterial collagen segments with incorporated hydroxyproline at Y position with consistent results.

Heterotrimeric collagens have not been as well researched as homotrimeric vertebrae collagens. It is difficult to achieve the correct stoichiometric ratio in solution with both synthetic peptides and recombinant collagen. Moreover, individual strands in the heterotrimer must be placed in the correct stagger (Figure 1). Drs. Boudko and Bachinger generated heterotrimeric recombinant collagen in *E. coli* using segments of α1 and α2 chains of Type 1 collagen by using the second non-collagenous domain (NC2) from Type IX Collagen to control trimerization (Boudko and Bächinger 2016; 2012). Stagger of the recombinant collagen could be controlled by using the 35 residue long NC2 domain of Type IX Collagen and led to the desired chains being in the leading, middle and lagging positions (Boudko and Bächinger 2016).

## Novel Biomaterials: Bioactive Hydrogels Based on Prokaryotic Collagen-Like Proteins

Collagen, the most abundant protein in the body, has been used as scaffolds for regeneration and repair of a variety of tissues, such as skin (Carver et al., 1995; Brown et al., 2002; Helary, Zarka, and Giraud-Guille 2012; Shokrgozar et al., 2012), cartilage (Li et al., 2020), bone (Salasznyk et al., 2004), vasculature (Stamati et al., 2014; Copes et al., 2019), and neural tissues (Suri and Schmidt 2010). Despite its broad use, mammalian collagens have significant limitations as scaffolds. As an animal-derived product, there is significant batch-to-batch variability due to harvest techniques, post-processing procedures, and storage conditions. Mammalian collagen products also suffer from a short product shelf life, diminishing bioactivity after processing, and potential immunogenicity (Cao and Xu 2008; Schmidt et al., 2016; Dhavalikar et al., 2020). Despite the aforementioned limitations, collagens contain substantial regenerative potential due to its native cell interactions, cell-responsive degradation, and tunable properties (Antoine et al., 2014). As such, there is an urgent need to find a replacement biomaterial that can provide these cellular cues while circumventing the limitations of current collagen scaffolds.

**FIGURE 3:**
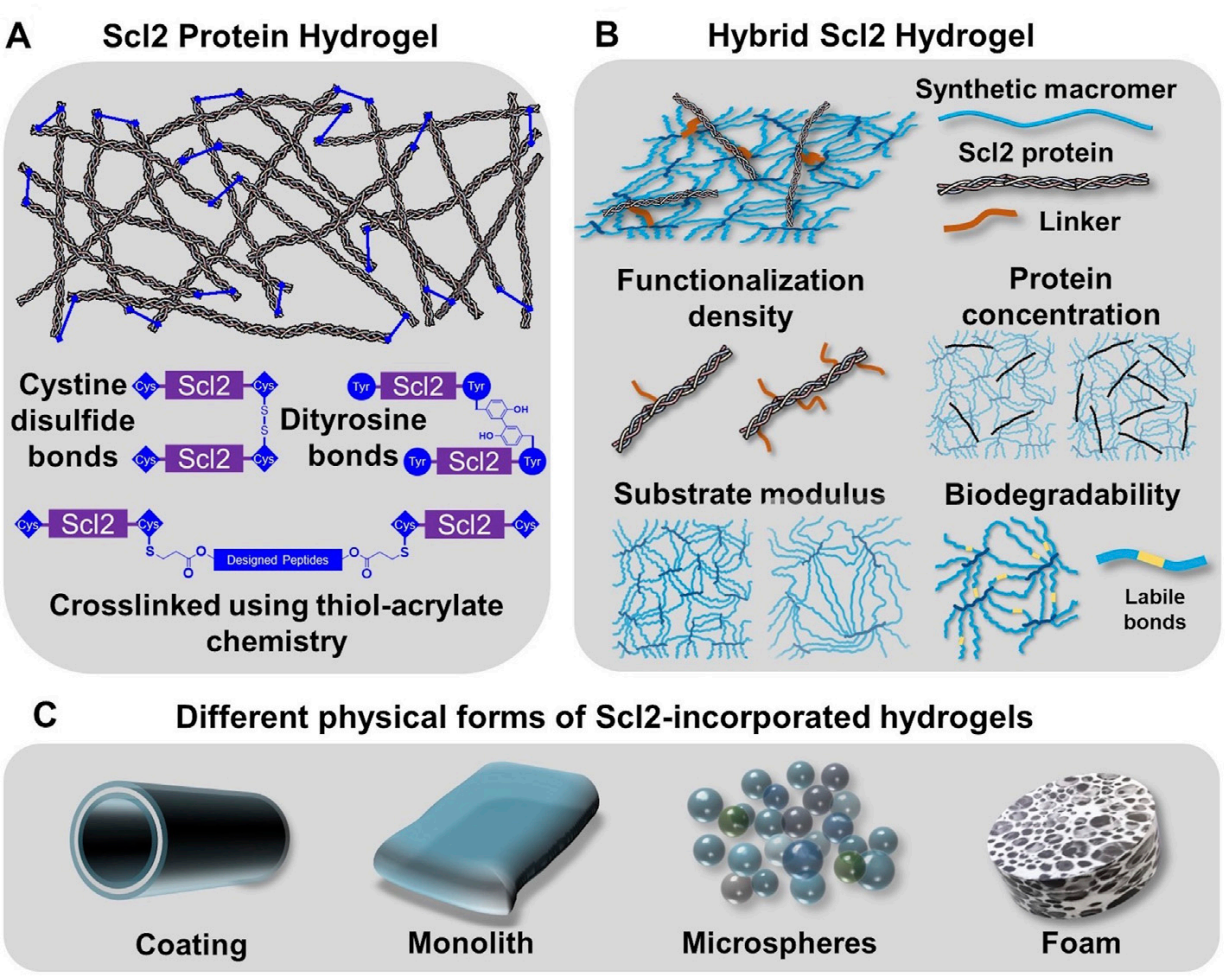
Incorporation of Scl2 proteins into hydrogels as alternative materials to animal-derived collagen matrices. **(A)** Scl2 protein hydrogel network with varied crosslinking modalities for enhanced gel stability. **(B)** Hybrid Scl2 hydrogels with the Scl2 protein anchored into a synthetic hydrogel network via a functionalization linker and the different parameters used to modulate hydrogel properties. **(C)** Different physcial forms of hybrid Scl2 hydrogels used in biomedical applications.

Previous sections have introduced the discovery of prokaryotic collagen-like proteins and the structural and functional resemblance to collagen that make it a strong candidate for bioengineering applications. Recombinant expression of prokaryotic collagen-like proteins provides a manufacturing process with minimal batch variability and scale-up potential. In addition, these prokaryotic collagen-like proteins generally have no inherent binding motifs for integrins and other extracellular matrix molecules which provides a unique opportunity for the engineering of specific cellular responses (Humtsoe et al., 2005; Kim et al., 2005; Xu et al., 2002). Selective insertion of multivalent binding sites for integrins and other molecules into the recombinant bacterial collagen has been used to generate targeted cell interactions to guide regeneration. In this section, we discuss strategies to design hydrogels based on recombinant bacterial collagen in biomedical applications, namely the Scl2.28 protein and Scl2 proteins derived from it (details included in the Supplementary Table S1). First, modification of Scl2 proteins to form hydrogels will be described. Hybrid Scl2 protein hydrogels will then be reviewed with the discussion of the effect of different fabrication parameters on the resultant cell-material interactions. Finally, the fabrication of the hybrid Scl2 hydrogels into different forms of hydrogel products will be described to demonstrate the versatility of this biomaterial platform (Figure 3).

### Scl2 Protein Hydrogels

Collagen matrices are typically crosslinked to form a functional gel and tune its physical properties and resorption rate. Given the similarities in structure and functional residues, prokaryotic collagen-like proteins like Scl2 proteins have the potential to form hydrogel network via the same chemical or enzyme-induced crosslinking methods. However, Peng et al. found that after freeze-drying and treatment with 1-ethyl-3-(3-dimethylaminopropyl)carbodiimide (EDC) for chemical crosslinking, Scl2 protein-based hydrogel sponges were unstable in water and resulted in structural disintegration (Peng et al., 2010). It was hypothesized that the instability of the EDC-crosslinking hydrogel was attributed to the smaller molecular weight of Scl2 protein as compared to collagen and fewer functional residues. A glutaraldehyde vapor was then applied as an alternative crosslinking mechanism. Although this generated stable hydrogels, there was limited control over crosslink density and hydrogel properties (Peng, Glattauer, and Ramshaw 2017). To resolve this problem, researchers introduced peptide sequences with functional residues to create additional crosslinking sites and stabilize the hydrogel network (Stoichevska et al., 2016; Stoichevska et al., 2017). Stoichevska et al. proposed introducing chemically functional residues to Scl2 proteins and forming crosslinks via oxidation (Stoichevska et al., 2016; Stoichevska et al., 2017). As shown in Figure 3A, cysteine or tyrosine residues were introduced to either or both C-terminal and N-terminal of the collagen-like domain. The hydrogel crosslinking was designed to form when cysteine residues form disulfide bonds or adjacent tyrosine residues form dityrosine bonds via photo-initiated oxidation. In this way, the collagen-like domain was unmodified and the specific binding sites for cell attachment via integrins and interactions with other extracellular matrix molecules were preserved. The results demonstrated that the tyrosine crosslinking resulted in gel formation while cysteine-based disulfide bonding did not form a hydrogel, although it significantly increased the number of intramolecular crosslinks (Winterbourn et al., 2004). This work also suggested different roles with intramolecular crosslinks forming small soluble aggregates and intermolecular crosslinks leading to hydrogel network formation. Similarly, Parmar et al. modified both the C- and N-termini of Scl2.28 proteins with an additional sequence, GGPCPPC (Parmar et al., 2016a). The cysteine residue was then designed to react with two different types of peptides that contained substrates for either matrix metalloproteinase 7 (MMP7) or aggrecanase. The linkage between the Scl2.28 proteins to these peptides was formed via thiol-acrylate “click” chemistry that required only mild reaction conditions and preserved protein bioactivity. This gave rise to a stable Scl2 protein network with MMP7- or aggrecanase-cleavable bonds, allowing for cell-responsive degradation of the hydrogel product after implantation.

In summary, typical collagen crosslinking methods are insufficient to make Scl2 protein hydrogels without additional modification of the protein to increase crosslinking sites due to its reduced length as compared to collagen. The incorporation of enzymatically labile sequences in the crosslinkers provides a well-defined hydrogel platform of Scl2 protein with high adaptability and an adjustable degradation profile. Although this provides a promising biomaterial platform, many of these studies did not demonstrate the ability of tuning the protein hydrogel mechanical properties. Furthermore, the use of enzymatically labile peptides produced by solid-phase synthesis reduces the cost-effectiveness of these protein hydrogels and creates challenges in large-scale production.

### Hybrid Scl2 Hydrogels

To expand the available physical properties to Scl2-based hydrogels, researchers created hybrid Scl2 hydrogels by incorporating Scl2 proteins into a synthetic hydrogel network (Cosgriff-Hernandez et al., 2010). Cosgriff-Hernandez et al. reported the first Scl2 protein functionalization method and incorporation into a hydrogel network to study the effect of different binding motifs for integrins α_1_β_1_ and α_2_β_1_ (Cosgriff-Hernandez et al., 2010). Cell culture studies with native and modified C2C12 cells demonstrated that an Scl2 protein modified with a collagen-derived GFPGER binding motif interacted with both α_1_β_1_ and α_2_β_1_ integrins; whereas, an Scl2 protein modified with a unique GFPGEN binding motif interacted only with α_1_β_1_ integrins. Endothelial cells adhered to and spread on both Scl2 proteins, but smooth muscle cells did not adhere to Scl2 proteins without the α_2_β_1_ integrin interaction (Cosgriff-Hernandez et al., 2010). In addition to selective cell interactions, these hybrid Scl2 hydrogels also provide a unique opportunity to decouple the Scl2 ligand presentation from other gel physical properties such as matrix stiffness and resorption rate. Scl2 proteins are first functionalized with a polyethylene glycol (PEG)-based linker that contains an acrylate or acrylamide group that allows it to be anchored into a PEG-based hydrogel (Figure 3B) (Cosgriff-Hernandez et al., 2010). PEG-based hydrogels have been widely used in numerous biomedical applications, such as cell-laden scaffolds, wound dressings, implant coatings, and interstitial tissue substitutes. The antifouling function of PEG hydrogels limits non-specific protein adsorption such that the protein presentation to cells is limited to the Scl2 protein (Chen et al., 2019). The insertion of Scl2 protein has minimal effect on the network formation of the polymeric hydrogel, allowing for the modulation of ligand presentation independent of substrate bulk properties. The following sections detail the effect of hydrogel properties on cell-material interactions.

## Ligand Concentration and Protein Functionalization Density

To understand the effect of ligand concentration and protein functionalization density on the cell-material interactions, Browning et al. compared cell behavior at different Scl2-2 protein (Supplementary Table S1) concentrations (Browning et al., 2014) and varying PEG linker densities on the Scl2 protein backbone (Browning et al., 2013). As the protein loading concentration increased, cell adhesion, proliferation, migration rate, and spreading increased at differing rates. By controlling the molar ratio of the linker to lysine residues to 0.1:1, 0.5:1, and 1:1, Scl2 proteins modified with integrin-binding motif GFPGER with varied functionalization densities were prepared and then incorporated into PEG diacrylate hydrogels. There was no significant difference in concentration of functionalized Scl2 proteins on swelling and mechanical properties of hydrogels. The initial incorporation and retention of the protein in the hydrogel was monitored for 6 weeks. The results demonstrated a decrease in protein content for all the functionalization densities with the proteins at the lowest functionalization density displaying the greatest protein loss (Browning et al., 2013). This was attributed to the hydrolytic degradation of the acrylate ester bonds and fewer linkages to the hydrogel network. Notably, reduced functionalization significantly improved cell adhesion and spreading to the bioactive hydrogels despite the reduced ligand concentration (Browning et al., 2013). It was hypothesized that the linker created steric hindrance around the ligand and reduced integrin binding. To overcome the steric hindrance caused by protein functionalization, Cereceres et al. redesigned the Scl2 protein to reduce steric hindrance to the integrin-binding site, GFPGER, by moving the reactive residues into the globular region and away from the integrin-binding sites (Cereceres et al., 2015). This newly engineered Scl2 protein hydrogel displayed a higher cell adhesion as compared to the original bioactive hydrogel.

## Substrate Modulus and Degradation Kinetics

Substrate stiffness has been identified as a key factor in directing cell adhesion, migration, and differentiation (Engler et al., 2006; Hynes 2009; O’Brien 2011; Lu, Weaver, and Werb 2012). Given this established mechanoresponsive behavior, cell behavior was characterized on Scl2 hydrogels of two different substrate moduli as controlled by PEG macromer molecular weight (10 vs 3.4 kDa) (Browning et al., 2014). Endothelial cell adhesion, migration, and spreading increased significantly on gels with a higher modulus. Unlike collagen hydrogels that often couple substrate stiffness and ligand presentation (e.g., increasing collagen concentration), the substrate modulus of these hydrogels is tunable independent of variations in protein functionalization and loading concentration (Browning et al., 2014). This uniquely permits the study of individual roles of substrate modulus and ligand presentation on integrin-mediated focal adhesion complex formation and the corollary effect on cell behavior.

In addition to substrate stiffness, these hybrid gels also offer a broader range of degradation kinetics to meet the needs of different applications. For example, a wound hydrogel scaffold can be designed to degrade to complement the rate of regeneration; whereas a hydrogel coating of cardiovascular devices should preserve the structure and functions over the life of the device (10–20 years) to provide long-term efficacy. Browning et al. developed a non-degradable hydrogel network of PEG-diacrylamide that replaced the hydrolytically labile ester bonds of PEG diacrylate (Browning et al., 2013). To increase the Scl2 protein retention in the hydrogel, a new biostable linker was designed to eliminate hydrolytically labile groups. This biostable linker markedly increased the retention of the protein in the hydrogel over 6 weeks while maintaining equivalent bioactivity. Researchers have also developed a degradable PEG-based hydrogel network for regenerative applications (Cereceres et al., 2015). In this work, more labile thio-β ester bonds were incorporated into the hydrogel network. Compositional control was used to generate a range of different degradation rates from days to months without changing the hydrogel bulk properties such as swelling or modulus. The degradable linkage can also be utilized to release the incorporated Scl2 protein locally.

## Hydrogel Fabrication

In addition to the hydrogel chemistry variables described above, Figure 3C illustrates some commonly utilized fabrication methods to generate hydrogel slabs, coatings, microspheres, and foams. Most commonly, PEG-Scl2 hydrogels (PEG-based hydrogels containing Scl2 proteins) are formed in molds via photopolymerization to generate bulk hydrogel slabs, tubes, or sheets (Cosgriff-Hernandez et al., 2010; Becerra-Bayona et al., 2018). Browning et al. developed a multilayer vascular graft by coating an electrospun polyurethane mesh with PEG-Scl2 hydrogels via UV-initiated polymerization with a tubular mold (Browning et al., 2012). However, difficulty in fabricating thin coatings and uneven coverage of the hydrogel on the mesh surface constrained the clinical application of the graft. Wancura et al. developed a new hydrogel coating fabrication method using redox-initiated polymerization and diffusion-mediated crosslinking (Wancura et al., 2020). The authors achieved the formation of uniform hydrogel coating layers with controlled thickness. In addition, this fabrication method allowed for the formation of multilayer hydrogel with distinct features and coating of the hydrogel on devices of complex structures and materials. PEG-Scl2 hydrogel microspheres were fabricated via a co-fluidics method to generate injectable wound dressings (Cereceres et al., 2015). Finally, Scl2 protein hydrogel sponges were fabricated and used to deliver hyaluronic acid and chondroitin sulfate and temporally control the sponge degradation to induce chondrogenesis for the articular cartilage repair (Parmar et al., 2016b).

In summary, Scl2 protein and hybrid PEG-Scl2 hydrogels have shown great potential to replace animal-derived collagen matrices. As a new biomaterial platform, Scl2 proteins offer significant advantages in selective cell interactions and tunable physical properties and forms. The next section will elaborate on the specific applications of these Scl2-based hydrogels in the regeneration and repair of different tissues.

## Application of SCL2 Proteins as Integrin-Targeting Materials

A key goal in biomaterial design for regenerative applications is directing cellular behavior. Integrin-targeting biomaterials provide a mechanism to mimic native cell-extracellular matrix interactions (Dhavalikar et al., 2020). Various extracellular matrix-derived proteins or peptide sequences have been utilized to target and bind specific integrins to direct cell proliferation, migration, and differentiation. The directed cell responses can facilitate numerous tissue repair processes, including ECM reorganization, angiogenesis, and tissue remodeling, to achieve improved and accelerated tissue repair or regeneration. Insertion of different binding motifs into the Scl2 protein backbone has been used to achieve selective integrin interactions (Stoichevska et al., 2016; Peng et al., 2014; An et al., 2016; 2013; 2014). As compared to collagen and other protein-based biomaterials that have multiple cellular interactions, these Scl2 proteins can be utilized to target specific cell behavior. In addition to providing improved medical devices, these new biomaterials also provide new tools to elucidate complex regenerative processes.

In this section, we will review recent studies applying these integrin-targeting materials based on Scl2 proteins to clinical applications. This includes thromboresistant coatings of cardiovascular devices, scaffolds for bone regeneration, chronic wound dressings, and matrices for cartilage repair.

### Thromboresistant Coatings for Cardiovascular Devices

Sustained thromboresistance has been a challenge in the field of cardiovascular devices that continues to limit clinical outcomes. The integrin-targeting nature of Scl2 proteins provides an opportunity to target endothelial cell interactions while limiting platelet attachment and activation. The hybrid Scl2 hydrogel combines the antifouling nature of PEG hydrogels for acute thromboresistance with the cell selectivity of the Scl2 proteins to promote endothelialization for sustained thromboresistance. Browning et al. investigated the ability of these hybrid Scl2 hydrogels to direct endothelial cell interactions (Browning et al., 2012; Browning et al., 2013; Browning et al., 2014). In these studies, researchers modulated protein-incorporated hydrogels by tuning the ligand concentration, adjusting protein functionalization density, changing the substrate stiffness, and constructing a biostable hydrogel network and functionalized linkage. Endothelial cell adhesion, spreading, and migration were then characterized, showing that the cell-material interaction was significantly affected by the accessibility and number of binding motifs on the Scl2 proteins and the bioactivity retention of proteins. Furthermore, PEG-Scl2 hydrogels showed minimal levels of platelet adhesion and activation indicating its thromboresistance (Browning et al., 2011).

To further understand the hemostatic regulation by different integrin-binding interactions, Post et al. fabricated bioactive hydrogels with different sets of integrin binding sites by incorporating different proteins, including Scl2_GFPGER_, collagen, and gelatin (Post et al., 2019). Combining the results of the flow cytometry and the human umbilical vein endothelial cell (HUVEC) attachment quantification after blocking integrins with antibodies, the researchers were able to demonstrate the specific integrin-binding sites of different proteins. The platelet activation study showed that the PEG-Scl2 hydrogel was the least pro-thrombotic and most anti-thrombotic among varied types of hydrogels, suggesting that GFPGER-binding integrins α_1_β_1_ and α_2_β_1_ supported a more anti-thrombotic HUVEC phenotype (Post et al., 2019). Platelet adhesion and activation results then verified that fewer platelets adhered to PEG-Scl2 hydrogel than the other types of protein-incorporated hydrogels. Additionally, HUVECs on PEG-Col hydrogel (PEGDA hydrogel incorporating natural collagen)and PEG-Scl2 hydrogel demonstrated similar PAC-1 expression levels to the PEGDA blank hydrogel that stimulated the minimal platelet activation (Post et al., 2019). These results suggest that integrins α_1_β_1_ and α_2_β_1_-mediated HUVEC responses can reduce the platelet adhesion and activation, and biomaterials targeting these integrins can potentially avoid deleterious coagulation cascade, thrombus formation, and vascular occlusion as previous studied (Radke et al., 2018). This highlighted the important roles of integrin-binding interaction and provided guidelines for biomaterial design, including the spatial and temporal exposure of specific integrin-binding sites to orchestrate target cellular processes at different tissue regenerative stages.

The integrin-binding ligand not only affects the adhesion, proliferation, and migration of human aortic endothelial cells, but also makes a difference in endothelial cell differentiational markers of VE-cadherin, PECAM-1, NOS3, TM, and E-selectin (Munoz-Pinto et al., 2015). These markers are correlated to the endothelization processes, such as the formation of the tight junction and the establishment of a confluent monolayer of cells. Both PECAM-1 and VE-cadherin expression and protein production levels of the endothelial cells cultured on the PEG-Scl2 hydrogels were not significantly different from that of PEG-Collagen hydrogels. The protein production of TM and E-selectin demonstrated no variance between hydrogels with the Scl2 proteins and collagens. However, PEG-Scl2 hydrogel did not induce the same expression level of NOS3 as the PEG-collagen hydrogel, which is significant for the vascular homeostasis. This was due to the other various binding sites on the natural collagen molecule that can promote the NOS3 expression and production. Still, the versatility of Scl2 proteins allowed for further modification that can implement the same effect on this specific marker expression, such as insertion of heparin-binding site. The studies were then extended to more endothelial cell phenotypes, that were endothelial progenitor cells (EOCs) and human umbilical vein endothelial cells (HUVECs) (Munoz-Pinto et al., 2015). Both gene expression and protein production results demonstrated a similar or improved endothelial maturation level of EOCs cultured on PEG-Scl2 hydrogel compared to that cultured on PEG-Collagen hydrogel. The PEG-Scl2 hydrogel had a similar or improved EOC homeostatic marker expression to the PEG-Collagen hydrogel. These results supported further exploration of PEG-Scl2 hydrogel as an advanced thromboresistant vascular graft coating. An increase in integrin binding sites of the protein significantly improved the cell adhesion and retention on the hydrogel substrate while maintaining a relatively high cell migration rate (Quiroz et al., 2018). EOCs cultured on PEG-Scl2 hydrogel with three integrin-binding sites displayed improved gene expression and protein production levels of intermediate differentiation markers compared to that of PEG-Collagen hydrogels. This work indicated that PEG-Scl2 hydrogels have the potential to improve endothelial cell differentiation with amplified integrin-mediated interactions when the cell adhesion, retention, and migration rate were suitable for the formation of a cellular monolayer.

**FIGURE 4:**
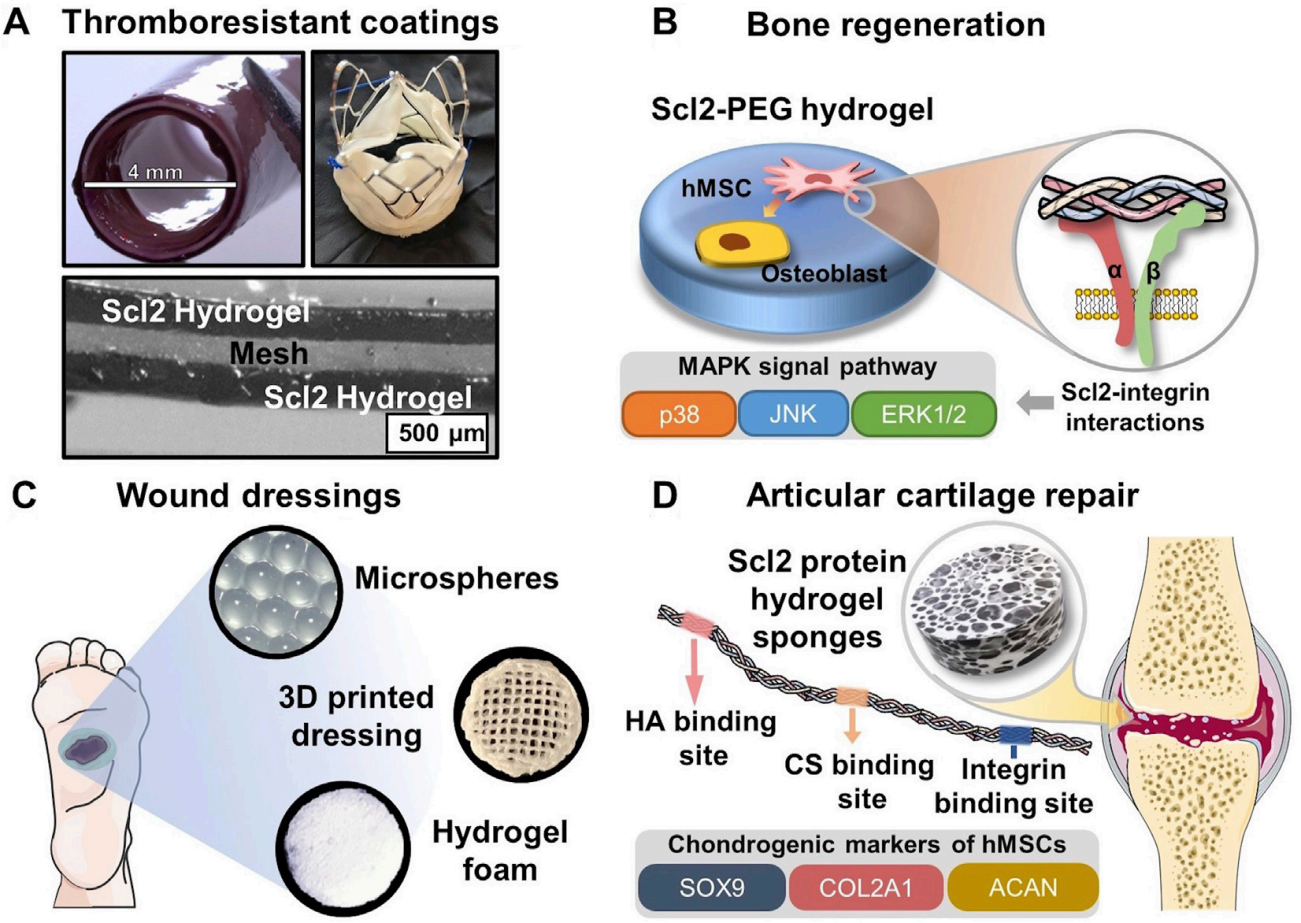
Biomedical applications of Scl2-containing biomaterials with targeted integrin interactions. **(A)** PEG-Scl2 hydrogel coatings of cardiovascular devices to enhance thromboresistance; **(B)** PEG-Scl2 hydrogel that revealed integrin-mediated hMSC osteogenic differentiation via the MAPK signal pathway **(C)** Different types of dressings containing Scl2 proteins to enhance chronic wound healing; **(D)** PEG-Scl2 protein hydrogel with binding sites for chondroitin sulfate and hyaluronic acid to improve cartilage repair.

Overall, these studies demonstrate that the incorporated Scl2 proteins can induce targeted integrin-mediated interactions with different types of endothelial cells to promote cell adhesion and migration, as well as hemostatic phenotypes. As shown in Figure 4A, the well-designed thromboresistant Scl2 protein hydrogel can be coated on the surface of cardiovascular grafts and heart valves with customized bioactivity and potential for sustained thromboresistance.

### Scaffolds for Bone Regeneration

Collagen I has been widely shown to support mesenchymal stem cell (MSC) osteogenic differentiation in both two-dimensional (2D) and three-dimensional (3D) environments (Donzelli et al., 2007; Chamieh et al., 2016; Chan et al., 2010; Lund et al., 2008; Schneider et al., 2010; Chiu et al., 2014; Somaiah et al., 2015). As such, the application of collagen-mimetic proteins to bone scaffold design has arisen. In Becerra-Bayona et al., Scl2 proteins were used to elucidate the impact of individual collagen motifs on associated cellular responses (Becerra-Bayona et al., 2018). As shown in Figure 4B, human MSCs (hMSCs) were cultured within PEG hydrogels containing either Scl2_GFPGER_ or Scl2_GFPGEN_ in the absence of osteogenic supplements. PEG-Scl2_GFPGER_ gels were associated with increased hMSC osterix expression, osteopontin production, and calcium deposition relative to the PEG-Scl2_GFPGEN_ gels (Becerra-Bayona et al., 2018). Results indicated that integrin α_2_ signaling may have an increased osteogenic effect relative to integrin α_1_. Since integrin α_1_β_1_ and α_2_β_1_ both trigger the MAPK pathway, ERK, JNK, and p38 activation were also assessed to evaluate potential mechanisms underlying observed responses. Inhibition results suggested that p38 activity triggered by integrin α_2_β_1_ but not α_2_β_1_ plays a key role in collagen-supported hMSC osteogenesis (Becerra-Bayona et al., 2018). Similarly, Reyes et al. found that surfaces coated with triple helical GFOGER (analogous to Scl2_GFPGER_ but without the flexibility afforded by recombinant expression) induced similar levels of expression of osteogenic markers runx2, osteocalcin, and bone sialoprotein by MC3T3-E1 as native collagen I (Reyes and García 2004). These triple helical GFOGER peptide coatings have further been passively adsorbed onto polymeric scaffolds and have also been found to significantly accelerate and increase bone formation in non-healing femoral defects compared to uncoated scaffolds and empty defects (Wojtowicz et al., 2010). Further studies with the more versatile Scl2 proteins will allow for improved design of collagen-based scaffolds.

### Chronic Wound Dressings

Natural collagen has been applied to different skin substitute products or wound dressings due to its compatibility and bioactive cues (Singer and Clark 1999). As an advantageous replacement for natural collagen, Scl2 proteins with binding sites of integrins α_1_β_1_ and α_2_β_1_ can direct keratinocyte migration and proliferation, ECM deposition, and wound contraction in the later stage of wound healing. As discussed in the previous section, Cereceres et al. redesigned Scl2 protein to have the integrin-binding motif, GFPGER, away from functionalization sites (Cereceres et al., 2015). By characterizing the relative α_1_ integrin-domain binding, the authors were able to prove that the relocation of integrin binding sites significantly reduced the steric blocking by the protein functionalization. As a result, the redesigned eCol_GFPGER_ hydrogel demonstrated a critical increase in fibroblast adhesion compared to the original Scl2_GFPGER_ hydrogel, suggesting increased integrin-mediated interactions. The well-defined hydrogel platform with the tunable presentation of integrin binding sites allows for a comprehensive investigation of the roles of different integrin-mediated signal pathways in wound healing. This contains a group of integrins that have been identified to influence different stages of wound healing, including formerly mentioned α_1_β_1_ and α_2_β_1_, as well as α_3_β_1_ and α_11_β_1_ (Koivisto et al., 2014; Larjava et al., 1996; Liu et al., 2010; Longmate and DiPersio 2014; Choma et al., 2007; Thannickal et al., 2003; Dhavalikar et al., 2020). Also, wound dressings of different forms (Figure 4C), including hydrogel microspheres, 3D-printed hydrogel dressing with hierarchical, and highly porous hydrogel foam, can be employed to deliver the Scl2 protein to the wound bed while providing moisture balance for improved healing potential.

### Matrices for Cartilage Regeneration

A series of studies were conducted by Parmar et al. on developing Scl2 protein sponges with inserted binding sites for hyaluronic acid (HA), chondroitin sulfate (CS), heparin, or integrin (Figure 4D) (Parmar et al., 2016a; Parmar et al., 2016b; Parmar et al. 2017). Specifically, the researchers introduced different cleavage linkages to the protein hydrogel sponge network to achieve temporal control over the hydrogel degradation, modulating the chondrogenesis of hMSCs (Parmar et al., 2016a). The results indicated that groups with a ratio of MMP7-cleavable and aggrecanase-cleavage linkages at 25:75 and 50:50 significantly upregulated chondrogenic expression levels, rendering the highest production levels of sulfated glycosaminoglycan (sGAG) and collagen. Additionally, the hMSC-laden protein hydrogel sponges of these two groups maintained their mechanical integrity and strength after 6 weeks, which was never seen in other degradable cartilage scaffold work. Another study from the group investigated the effect of HA and chondroitin sulfate binding site ratio on cell responses and chondrogenesis (Parmar et al., 2016b). *In vitro* characterization identified that the hydrogel foam groups with HA-Scl2 and HA:CS (75:25)-Scl2 proteins (Supplementary Table S1) had drastically upregulated expression levels of chondrogenesis markers COL2A1, ACAN, and SOX9 with greatest sGAG and collagen production. Importantly, these works have provided a versatile Scl2 protein hydrogel platform with tunable binding sites and adjustable degradation profiles, demonstrating potential applications in a broader tissue engineering context. However, although the integrin-binding sites were inserted in the employed Scl2 protein, the authors had not identified the integrins of interest in chondrogenic differentiation of hMSCs. Other research indicated that β_1_ integrins, including α_1_β_1_, α_2_β_1_, and α_3_β_1_ of collagen receptors, were activated during chondrogenic differentiation (Takahashi et al., 2003; Goessler et al., 2008). Thus, a future direction of Scl2 protein hydrogel application in articular cartilage repair can be the investigation of different integrin-mediated cell behaviors by Scl2 proteins with specific binding sites.

This section reviewed several applications of Scl2 protein hydrogels in repairing and regenerating damaged tissues of the vasculature, bone, skin, and articular cartilage. With highly tunable mechanical properties and customized bioactivity, these Scl2-based hydrogels demonstrated the versatility of this new biomaterial platform. Furthermore, this novel class of biomaterials is capable of extending our understanding of the biological processes in cellular behaviors and tissue recovery and guiding the future biomaterial design for more advanced solutions in regenerative medicine.

## Current Challenges and Future Perspectives

The selective insertions of specific integrin-binding motifs into Scl2 proteins provided numerous opportunities for advancing the understanding of integrin-ligand interactions, integrin-mediated signal pathways, and resultant cellular behaviors. Native collagens have a collection of integrin binding sites that can be countered, compounded, or have synergetic effects after interacting with specific integrins. With no inherent binding motif, Scl2 proteins can be inserted with one or more types of ligands as designed. Previous studies have utilized engineered Scl2 proteins to facilitate the investigation of multiple critical integrin-mediated cell behaviors in bone regeneration, endothelialization, and hemostatic regulation with vascular devices (Browning et al., 2012; Becerra-Bayona et al., 2018; Post et al., 2019; Wancura et al., 2020). Moreover, isolated fundamental integrin-ligand interactions in re-epithelialization, ECM redeposition, and wound contraction demand much attention and further probing for advanced wound healing modalities. It is also necessary to identify and isolate fundamental integrin interactions that play central roles in the chondrogenic differentiation of hMSCs and the essential glycosaminoglycan network reconstruction. Researchers have also discovered that varied types of cells express distinct sets of integrins, and these cells demonstrate different attachment affinities to other binding motifs (Cosgriff-Hernandez et al., 2010; Post et al., 2019). Hence, Scl2 proteins with inserted binding motifs allow for possible mapping of the relative affinities between different combinations of integrins and binding motifs in the context of multiple cells active in a particular circumstance or different cell phenotypes during differentiation. Substrates coated with recombinant Scl2 proteins can select cells from a co-culture system or target cells at defined differentiation stages. Regardless, recombinant bacterial collagens with inserted integrin-binding sites offer the opportunities to compare different integrin-ligand binding affinities, revealing the roles of isolated integrin-ligand interactions in the given tissue engineering field guide the biomaterial design for enhanced clinical outcomes.

The previous sections show examples of Scl2 proteins to develop biomaterials that guide integrin-mediated cellular activities for advanced tissue engineering. Therefore, the ligand presentation to the target cell population is essential for biomaterial design. It has been demonstrated that a change in the integrin ligand number of Scl2 proteins could result in different cell adhesion, migration rate, and marker expression levels (Quiroz et al., 2018). Also, the designed incorporation of recombinant bacterial collagens into highly tunable synthetic polymer hydrogels enabled the improvement and modulation of ligand exposure to the integrins (Cosgriff-Hernandez et al., 2010; Browning et al., 2011; Browning et al., 2013; Browning et al., 2014). However, cells interact with all the components of their surrounding environment dynamically, so spatial and temporal controls of specific integrin interaction play pivotal roles in understanding cell behaviors and directing tissue engineering. At the subcellular level, grouping multiple ligands together in islands can effectively promote integrin clustering and amplify targeted cell responses. The substrate mechanical properties, surface topography, distribution, and density of ligands can also be modulated to render different integrin-ligand binding modalities, leading to cell adhesion, migration, differentiation, and ECM production changes. In addition, there is a gap between 2D and 3D cell culture systems as 3D culturing has increased surface area for integrin binding and mimics the cell native niche with a dynamic interface. Integrin clusters are more prominent with longer lifetimes in 3D environments (Lepzelter et al., 2012). Cutting-edge nanopatterning techniques allow for a refined design of the biomaterial surface with a much higher resolution and versatile geometries and demonstrate a great promise in controlling the ligand accessibility to targeted cells. Substrates with temporally controlled degradation profiles induced by hydrolysis or enzyme cleavage potentiate the sequential exposure of different integrin-binding sites on the embedded Scl2 proteins to modulate specific cell behaviors in different regenerative stages. Biomaterial design and development have been advanced by the synthetic polymer science of growing versatility and continuously progressing fabrication techniques. However, there are remaining critical challenges in translating *in vitro* findings to *in vivo* results and applying the materials or devices to damaged tissues. For evaluating the effect of a chosen integrin interaction with its binding site, transgenic mice with ablation or deletion of targeted integrin genes have been studied *in vivo*. However, they are limited by unknown outcomes caused by mutagenesis. Also, fundamental biological factors must be considered in the context of a dynamic *in vivo* environment. Key players, including multiple other cells, plenty of growth factors, and remodeling ECM, can engender ambiguous or unreliable conclusions by compensating or competing with the ligands presented by the biomaterial. Researchers must make scrupulous assumptions and analyze the outcomes critically to avoid false conclusions in elucidating the roles of integrins in the cell response to designed biomaterials.

Animal-derived collagens have been widely used in biomedical applications due to their biocompatibility, biodegradability, and easy accessibility. However, its use has been hindered by batch-to-batch variability and limited potential for production scaling-up. The discovery of prokaryotic collagen-like proteins has provided opportunities to meet natural collagens’ structural and functional needs in various applications while improving production rates and reducing product variability. With selectively inserted binding sites, prokaryotic collagen-like protein has been shown in multiple publications to interact with extracellular matrix molecules and bind to targeted integrins, leading to appropriate cellular responses. This technology provides a versatile platform to investigate collagen binding sites, characterize their interaction with extracellular matrix proteins, elucidate specific cellular responses to the prokaryotic collagen-like protein-incorporated material, and guide the design of advanced biomaterials for tissue engineering.
