# Supplementary material for: Prokaryotic Collagen-Like Proteins as Novel Biomaterials
Source: Front Bioeng Biotechnol. 2022 Mar 17;10:840939. doi: 10.3389/fbioe.2022.840939 (PMC8968730; doi:10.3389/fbioe.2022.840939)
Supplement: Supplementary file 1 [file Table1.docx]

**Supplementary Table S1.** Different types of Streptococcal collagen-like proteins (Scl2 proteins) and their specific binding sites for integrins or other molecules

| Scl2^1^ Protein | Binding sites (receptor molecules) | References |
| --- | --- | --- |
| Scl2.28, Scl2-1 | Blank | (Xu et al. 2002; Cosgriff-Hernandez 2010) |
| Scl2-2, Scl2_GFPGER_, Designer Collagen Scl2-2 (DC2-1X)^2^ | GFPGER (integrins α_1_β_1_ and α_2_β_1_) | (Cosgriff-Hernandez 2010; Browning 2012; 2013; 2014; Cereceres 2015; Post 2019; Munoz-Pinto 2015; Munoz‐Pinto 2017; Becerra‐Bayona 2018; Diaz Quiroz et al. 2018) |
| Scl2-3, Scl2_GFPGEN_ | GFPGEN (integrin α_1_β_1_) | (Cosgriff-Hernandez 2010) |
| eCol_GFPGER_^3^ | GFPGER (integrins α_1_β_1_ and α_2_β_1_) | (Cereceres 2015) |
| DC2-2X^2^ | GFPGER (integrins α_1_β_1_ and α_2_β_1_) | (Diaz Quiroz et al. 2018) |
| DC2-3X1^4^ | GFPGER (integrins α_1_β_1_ and α_2_β_1_) | (Diaz Quiroz et al. 2018) |
| DC2-3X2^4^ | GFPGER (integrins α_1_β_1_ and α_2_β_1_) | (Diaz Quiroz et al. 2018) |
|  |  |  |
| V-CL^5^ | Blank | (Peng et al. 2014) |
| V-CL(H) | GRPGKRGKQGQK (Heparin) | (Peng et al. 2014) |
| V-CL(I) | GFPGER (integrins α_1_β_1_ and α_2_β_1_) | (Peng et al. 2014) |
| V-CL(HI) | GRPGKRGKQGQK (Heparin), GFPGER (integrins α_1_β_1_ and α_2_β_1_) | (Peng et al. 2014) |
| Scl2 protein that contains H-binding, I-binding, and HA-binding peptide sequences | GRPGKRGKQGQK (Heparin), GFPGER (integrins α_1_β_1_ and α_2_β_1_), RYPISRPRKR (hyaluronic acid) | (Parmar, Skaalure, et al. 2016) |
| HA-Scl2 | RYPISRPRKR (hyaluronic acid) | (Parmar, St-Pierre, et al. 2016) |
| CS-Scl2 | YKTNFRRYYRF (chondroitin sulfate) | (Parmar, St-Pierre, et al. 2016) |
| VCL^5^-ST_6_ (ST = silk sequence GAGAGS) | Blank | (An et al. 2013) |
| VCL-Fn-ST_6_ | GLPGQRGER (fibronectin) | (An et al. 2013) |
| VCL-Int-ST_6_ | GFPGER (integrins α_1_β_1_ and α_2_β_1_) | (An et al. 2013) |
| VCL-Int-Fn-ST_n_  (n = 3, 6, 9) | GLPGQRGER (fibronectin), GFPGER (integrins α_1_β_1_ and α_2_β_1_) | (An et al. 2013) |

1 Scl2: Streptococcal collagen-like proteins

2 DC2-nX (n = 1 or 2): Designer collagen (Scl2-2) with one or two integrin-binding site(s)

3 eCol_GFPGER_: Engineered collagen, a modified Scl2_GFPGER_

4 DC2-3Xm (m = 1 or 2): Designer collagen (Scl2-2) with three integrin-binding sites; different “m” number stands for different insertion location for the third integrin-binding site

5 V-CL/VCL: Collagen-like protein with a globular region (V)

**References:**

An, Bo, T. M. DesRochers, G. Qin, X. Xia, G. Thiagarajan, B. Brodsky, and D. L. Kaplan. 2013. “The Influence of Specific Binding of Collagen-Silk Chimeras to Silk Biomaterials on HMSC Behavior.” *Biomaterials* 34 (2): 402–12. https://doi.org/10.1016/j.biomaterials.2012.09.085.

Becerra‐Bayona, S.M., Guiza‐Arguello, V.R., Russell, B., Höök, M. and Hahn, M.S. 2018. “Influence of Collagen‐based Integrin Α1 and Α2 Mediated Signaling on Human Mesenchymal Stem Cell Osteogenesis in Three Dimensional Contexts.” *Journal of Biomedical Materials Research Part A* 106 (10): 2594–2604.

Browning, M.B., Dempsey, D., Guiza, V., Becerra, S., Rivera, J., Russell, B., Höök, M., Clubb, F., Miller, M., Fossum, T. and Dong, J.F. 2012. “Multilayer Vascular Grafts Based on Collagen-Mimetic Proteins.” *Acta Biomaterialia* 8 (3): 1010–21.

Browning, M.B., Guiza, V., Russell, B., Rivera, J., Cereceres, S., Höök, M., Hahn, M.S. and Cosgriff-Hernandez, E.M. 2014. “Endothelial Cell Response to Chemical, Biological, and Physical Cues in Bioactive Hydrogels.” *Tissue Engineering Part A* 20 (23–24): 31303141.

Browning, M.B., Russell, B., Rivera, J., Höök, M. and Cosgriff-Hernandez, E.M. 2013. “Bioactive Hydrogels with Enhanced Initial and Sustained Cell Interactions.” *Biomacromolecules* 14 (7): 2225–33.

Cereceres, S., Touchet, T., Browning, M.B., Smith, C., Rivera, J., Höök, M., Whitfield-Cargile, C., Russell, B. and Cosgriff-Hernandez, E. 2015. “Chronic Wound Dressings Based on Collagen-Mimetic Proteins.” *Advances in Wound Care* 4 (8): 444–56.

Cosgriff-Hernandez, E., Hahn, M.S., Russell, B., Wilems, T., Munoz-Pinto, D., Browning, M.B., Rivera, J. and Höök, M. 2010. “Bioactive Hydrogels Based on Designer Collagens.” *Acta Biomaterialia* 6 (10): 3969–77.

Diaz Quiroz, Juan Felipe, Patricia Diaz Rodriguez, Josh D. Erndt-Marino, Viviana Guiza, Bailey Balouch, Tyler Graf, William M. Reichert, Brooke Russell, Magnus Höök, and Mariah S. Hahn. 2018. “Collagen-Mimetic Proteins with Tunable Integrin Binding Sites for Vascular Graft Coatings.” *ACS Biomaterials Science & Engineering* 4 (8): 2934–42. https://doi.org/10.1021/acsbiomaterials.8b00070.

Munoz‐Pinto, D.J., Erndt‐Marino, J.D., Becerra‐Bayona, S.M., Guiza‐Arguello, V.R., Samavedi, S., Malmut, S., Reichert, W.M., Russell, B., Höök, M. and Hahn, M.S. 2017. “Evaluation of Late Outgrowth Endothelial Progenitor Cell and Umbilical Vein Endothelial Cell Responses to Thromboresistant Collagen‐mimetic Hydrogels.” *Journal of Biomedical Materials Research Part A* 105 (6): 1712–24.

Munoz-Pinto, D.J., Guiza-Arguello, V.R., Becerra-Bayona, S.M., Erndt-Marino, J., Samavedi, S., Malmut, S., Russell, B., Höök, M. and Hahn, M.S. 2015. “Collagen-Mimetic Hydrogels Promote Human Endothelial Cell Adhesion, Migration and Phenotypic Maturation.” *Journal of Materials Chemistry B* 3 (40): 7912–19.

Parmar, Paresh A., Stacey C. Skaalure, Lesley W. Chow, Jean-Philippe St-Pierre, Violet Stoichevska, Yong Y. Peng, Jerome A. Werkmeister, John A. M. Ramshaw, and Molly M. Stevens. 2016. “Temporally Degradable Collagen–Mimetic Hydrogels Tuned to Chondrogenesis of Human Mesenchymal Stem Cells.” *Biomaterials* 99 (August): 56–71. https://doi.org/10.1016/j.biomaterials.2016.05.011.

Parmar, Paresh A., Jean-Philippe St-Pierre, Lesley W. Chow, Jennifer L. Puetzer, Violet Stoichevska, Yong Y. Peng, Jerome A. Werkmeister, John A. M. Ramshaw, and Molly M. Stevens. 2016. “Harnessing the Versatility of Bacterial Collagen to Improve the Chondrogenic Potential of Porous Collagen Scaffolds.” *Advanced Healthcare Materials* 5 (13): 1656–66. https://doi.org/10.1002/adhm.201600136.

Peng, Yong Y., Violet Stoichevska, Kristin Schacht, Jerome A. Werkmeister, and John A. M. Ramshaw. 2014. “Engineering Multiple Biological Functional Motifs into a Blank Collagen-like Protein Template from Streptococcus Pyogenes.” *Journal of Biomedical Materials Research Part A* 102 (7): 2189–96. https://doi.org/10.1002/jbm.a.34898.

Post, A., Isgandarova, S., Martinez-Moczygemba, M., Hahn, M., Russell, B., Hook, M. and Cosgriff-Hernandez, E. 2019. “Elucidation of Endothelial Cell Hemostatic Regulation with Integrin-Targeting Hydrogels.” *Annals of Biomedical Engineering* 47 (3): 866–77.

Xu, Yi, Douglas R. Keene, Janusz M. Bujnicki, Magnus Höök, and Slawomir Lukomski. 2002. “Streptococcal Scl1 and Scl2 Proteins Form Collagen-like Triple Helices.” *Journal of Biological Chemistry* 277 (30): 27312–18. https://doi.org/10.1074/jbc.M201163200.
